# A Comparative Thermoacoustic Insulation Study of Silica Aerogels Reinforced with Reclaimed Textile Fibres: Cotton, Polyester and Wool

**DOI:** 10.3390/gels9070548

**Published:** 2023-07-05

**Authors:** Teresa Linhares, Vitor H. Carneiro, Maria T. Pessoa de Amorim, Luisa Durães

**Affiliations:** 1 University of Coimbra, CIEPQPF, Department of Chemical Engineering, 3030-790 Coimbra, Portugal; 2 2C2T-Centre for Textile Science and Technology, University of Minho, Campus of Azurém, 4800-058 Guimarães, Portugal; mtamorim@det.uminho.pt; 3 CMEMS, University of Minho, Campus of Azurém, 4800-058 Guimarães, Portugal; vitorcarneiro@utad.pt; 4 Department of Engineering, Universidade de Trás-os-Montes e Alto Douro, Quinta de Prados, 5001-801 Vila Real, Portugal; 5 Metrics, University of Minho, Campus of Azurém, 4800-058 Guimarães, Portugal

**Keywords:** silica aerogel composites, aerogel reinforcement, thermoacoustic insulation, textile industry, reclaimed fibres

## Abstract

Silica aerogels are highly porous materials with exceptional thermal insulation performance. They become even more attractive if combined thermal and acoustic insulation is achieved. Silica aerogel composites reinforced with fibres are an ingenious way to surpass the fragility stemmed from the aerogel’s intrinsic porosity, and textile fibres are good sound absorption materials. Reclaimed fibres are a relatively low-cost feedstock and were obtained in this work exclusively through mechanical processes from textile wastes, thus promoting the concept of circular economy, namely for cotton, polyester and wool fibres. These reclaimed fibres were used as reinforcement matrices for silica aerogel composites obtained from sol–gel transformation of tetraethyl orthosilicate and isobutyltriethoxysilane/or vinyltrimethoxysilane precursors and dried at ambient pressure after silylation. Silica aerogel composites reinforced with reclaimed cotton fibres had the best sound absorption coefficient (a peak value of 0.89), while the polyester-reinforced composite exhibited the lowest thermal conductivity (*k =* ~24 mW m^−1^ K^−1^, *Hot Disk*). The better combined results on thermal and acoustic insulation were achieved by the wool-reinforced composites. The thermal conductivity values were less than 27 mW m^−1^ K^−1^, and the sound absorption coefficient achieved a peak value of 0.85. Therefore, the aerogel composites developed here can be selected for thermal or/and acoustic barriers by choosing a suitable type of fibre. Their design and preparation protocol followed environmental-friendly and cost-effective approaches.

## 1. Introduction

Silica aerogels are attractive materials due to their unusual features, with emphasis on the extreme porosity, putting them at the lower end of the density range for solid man-made materials [1] and giving them a remarkable insulation ability [2]. However, the highly porous ceramic nanostructure that grants such uniqueness is also responsible for their intrinsic brittleness, which makes their processing and handling difficult [3]. Therefore, common daily applications are limited. Silica aerogel composites reinforced with fibres are an ingenious way to surpass the fragility stemmed from the aerogel’s intrinsic porosity, thus allowing an easier handling [4]. Several types of fibres are being used, ranging from natural to man-made fibres, with specific features that are often transferred to the silica aerogel composites. 

The world production of textile fibres increased from 8.4 to 14.3 kg per person per year for 1975 and 2021, respectively [5]. Despite the current uncertainty, a continuous rising trend is expected [5], posing environmental issues. Waste and leftovers are intrinsic to any industrial process, and such high levels of production inevitably lead to huge amounts of industrial textile residues. According to a recent European Parliament briefing report, the leftover fabric from the cutting rooms of the clothing industry accounts for around 20% of the textile materials’ consumption [6]. These residues, often with no other end use, are included in the cost of the products, ultimately paid by the consumers, while contributing to the Earth’s ecological unbalance. The reuse of wasted materials is known as a way to reduce the carbon footprint of the textile industry, focusing on the zero-waste concept [7].

The so-called reclaimed fibres are obtained from the textile wastes through mechanical processes [8] and can be a proper material to strengthen silica aerogels [4]. The wastes are broken down, mainly due to the abrasion of steel pins on a drum surface, by taking several passages until the material becomes single fibres again. The process preserves their chemical properties, but the reclaimed fibres tend to be smaller than the initial ones [8].

Research on the reinforcement matrices with fibres recovered from wastes is starting to emerge [9,10,11], addressing the concerns of the escalating increase in natural resources’ depletion.

The current insulation market materials are mainly blankets of inorganic fibres (glass or mineral wool) and expanded polystyrene foams, but many other conventional materials are also used [12]. Depending on the specificities of the structures, thermal insulation barriers are often classified as inorganic, organic or composite materials [13]. Some examples include the following: inorganic-based foams (from glass, concrete, or clay); synthetic foams derived from organic polymers (polyurethane or extruded polystyrene); fibre battings (sheep’s wool or man-made fibres); and composite materials (gypsum-based or wood panels) [13]. All those conventional insulation materials are economically affordable, but the insulation performance is generally achieved with thicker materials [14]. On the other hand, silica aerogel insulation materials can achieve better performance levels at quite reduced thicknesses [14,15] but are not yet affordable materials [14,16]. However, the aerogel thermal insulation market is expected to grow at a higher pace, driven by the increasing need for efficient insulation materials to foster energy savings [12].

Silica aerogels are an emerging market product, known by their *sui generis* mesoporous structure, making them ideal thermal insulators. The smaller dimension of pores compared to the mean free path of the air inhibits the movement of the air molecules and effectively prevents heat transfer [17]. Many other applications, not yet so disseminated, also benefit from their unique set of features, for example, filters, dielectric materials, biomedical devices or acoustic barriers [4,18]. Sound absorption materials classically rely on increased thickness when it comes to the frequency range below 2 kHz [19], which can be unfavourable if space is a concern. In opposition, silica aerogel composites can be effective sound absorbers at reduced thicknesses [17,20]. There is a vast application potential for silica aerogel composites in the construction of aircraft, spacecraft or ships. Apart from the saving of useable and high-value space, these are lightweight materials that also contribute to energy savings, an increasing demand particularly in the transportation sector [21]. Ideally, the reinforcement matrices shall not deteriorate the quite low intrinsic thermal conductivity of silica aerogels. On the other hand, according to a study sponsored by the European Union [22], the use of by-products, such as textile fibres, is encouraged in the development of sustainable sound absorbers. Combined synergies of different functionalities can be conceived, dependent on the features of the fibres used for reinforcement, as proposed in our previous work [9].

Most of the textile fibres can compete with the traditional sound absorbers, particularly at mid and high frequencies [22]. According to the literature research on the acoustic properties of natural fibres, the sound absorption coefficient of a pad of raw cotton fibres equalled the sound absorbers made of rock wool or fibre glass, all materials having the same thickness [23]. The irregularities present on the fibre surface, such as slits or cavities, are attributes that can enhance the sound absorption [22], and the ribbon-like shape of cotton fibres, with convolutions at regular intervals, or the scaled surface of wool fibres fulfil that prerequisite. Theoretically, the severe mechanical treatments that reclaimed fibres endure to convert the scraps of fabric into single fibres might be an additional benefit, because making them shorter and often thinner after being teared apart leads to an increased surface area, which is a way to potentiate the interaction between sound waves and the fibres [24,25].

A comparison on the insulation performance of silica aerogels reinforced with reclaimed textile fibres was the inspiration for this new work. The concept of sustainable development urges us to rethink the conception and development of market products, while the public awareness on the long-term ecological balance drives an increasing number of consumers to make environmentally friendly choices. 

Reclaimed cotton, polyester and wool fibres, obtained from textile industry leftovers, were used as the reinforcement matrices, after being carded to align the fibres in mats of regular thickness, hence with feasible manufacturing techniques, foreseeing the potential scale-up and future commercialization. Thermal insulation and sound absorption ability are complex functions of intrinsic material properties, such as density, and extrinsic properties, such as surface roughness or sample thickness [17], and the features of the reinforcement matrix play a key role. The main purpose of this study was to spot the best fitted properties of the developed composites in terms of the effect of the reclaimed fibres used as the reinforcement matrix, aiming for them to perform as insulation barriers, both thermal and acoustic.

## 2. Results and Discussion

The first step towards a better understanding of the final properties of the silica aerogel composites was the identification and characterization of the reinforcement matrices. Thereafter, the insulation performance of the composites, both thermal and acoustic, was evaluated. Other studies, namely SEM analysis and analysis of the mechanical properties of the composites, were carried out.

### 2.1. Characterization of Reclaimed Fibres

The reclaimed fibres of cotton, polyester and wool were characterized in terms of length, diameter, level of damage and chemical identification by FTIR analysis. 

#### 2.1.1. Length

**Figure 1:**
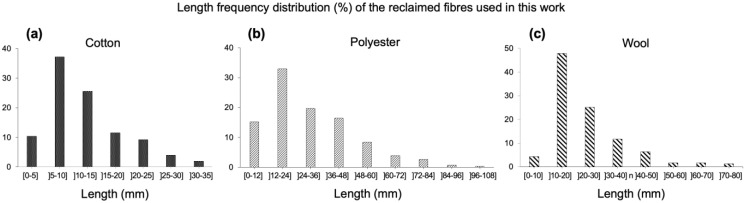
Fibre length frequency distribution for reclaimed fibres used in this work: (**a**) cotton, (**b**) polyester, (**c**) wool.

The length of virgin natural fibres is highly variable, because it is affected by genetic traits, growing conditions and pre-treatment processes [25,26]. Therefore, the characterization of natural fibres in terms of length is commonly described within a range [26]. On the other hand, man-made fibres can be produced as a continuous filament or as a staple fibre of precise length, mainly to produce textile yarns with an intimate blend of other staple fibres, by combining the desirable properties of each fibre [27]. The length frequency distributions of reclaimed cotton, polyester and wool fibres can be seen in Figure 1a, Figure 1b and Figure 1c, respectively. All the reclaimed fibres’ distributions deviate from the symmetric bell-shape distribution of the natural fibres, displaying a similar skewed distribution towards the smaller length values.

The length frequency distribution curve of virgin combed cotton fibres approximates from the normal, thus symmetric, distribution [28]. The length of virgin fibres from one of the most representative cotton species (*Gossypium hirsutum*) ranges between 25 and 29 mm [25]; other cotton species can yield fibres with lengths as low as 15 mm or higher than 50 mm [29]. According to the cotton terminology, the definition of “short fibre” is applied for lengths lower than 12.7 mm [28,30], and short fibres negatively affect the quality and uniformity of textile yarns in the spinning industry [31]. As can be seen in Figure 1a, reclaimed cotton fibres encompass a high share of short fibres. The average length value is as low as 12.4 ± 6.8 mm, a consequence of the harsh mechanical processes. The high value of standard deviation reveals a high variation of measurements. Moreover, ~60% of the reclaimed cotton fibres have a length smaller than 12.7 mm, denoting inadequacy for the conventional spinning processes [31]. But they can, however, be suitable for nonwoven fabric production [8], that in turn can be used in value-added materials. 

The original polyester staple format is cut at a required and/or customized length, generally higher than 32–34 mm [32,33]. Here, the high content of smaller length fibres is an indicator of the mechanical processes to which the polyester textile structures were subjected to become individual fibres again. The average length of the reclaimed polyester fibres was recorded as 30.3 ± 18.2 mm (see Figure 1b), a longer length than cotton fibres (see Figure 1a). The difference can be explained by the plausible higher original length of the virgin polyester fibres, but mainly by the high tenacity (more than double) and increased elasticity of polyester (20% vs. 5%) when compared to the cotton fibres [34]. These features impart good resiliency to the polyester fibres, protecting them from major damaging levels.

The length frequency distribution curve of virgin combed wool is very close to the normal distribution, yet with most frequent scores slightly towards to the longer lengths [35]. In terms of fibre length, sheep’s wool can vary between 30 and 55 mm; but depending on the time between the shearings of the sheep, fibres of 80 mm also exist [36]. The reclaimed wool fibres used in this work have an average length value of 23.4 ± 13.2 mm (Figure 1c), with more than 50% of fibres ≤ 20 mm, a clear outcome of the mechanical stresses the fibres endured. When compared to the length of reclaimed cotton fibres (see Figure 1a), the larger mean length value can be attributed to the greater elasticity and original length of wool fibres. When compared to the length of reclaimed cotton fibres (in Figure 1a), the highest mean value for the length of reclaimed wool fibres (in Figure 1c) can be attributed to their longer original length but also to their outstanding elasticity [37].

#### 2.1.2. Diameter and Damage Level of the Reclaimed Fibres

**Figure A1:**
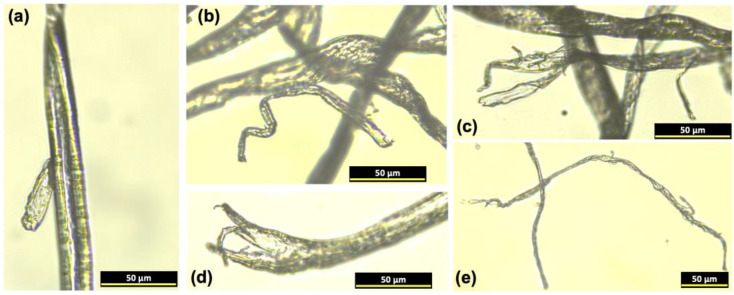
Damage of reclaimed cotton fibres: (**a**,**b**) ripped fragments; (**c**,**d**) shattered fibres; (**e**) split apart throughout the fibre longitudinal axis.

By using a fluorescence microscope, cotton fibres were clearly identified by the typical convolutions and have diameters that vary from less than 12 µm to more than 25 µm. The level of damage that contributes to increased surface area, which is relevant to enhance the sound absorption coefficient properties, as mentioned before, is presented in Appendix A, Figure A1. The harsh mechanical tensions caused ripped fragments and shattered and split-apart fibres throughout the longitudinal axis.

**Figure A2:**
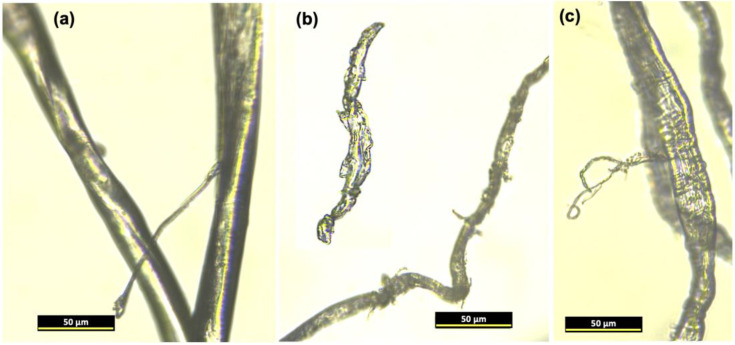
Damaged reclaimed polyester fibres: (**a**–**c**) tiny detached parts of fibres and (**b**) scratched fibre surface.

The circular cross-section is characteristic of most man-made fibres, and the polyester fibres had diameter values ranging from ~10 to 30 µm. The level of damage of these reclaimed fibres is not as severe as the one observed for cotton fibres, which can be explained by the higher resiliency of polyester fibres to face the applied forces. Apart from the surface scratches and broken fibres, other flaws encountered refer to tiny segments that were ripped out from fibres, as depicted in Appendix A, Figure A2.

**Figure A3:**
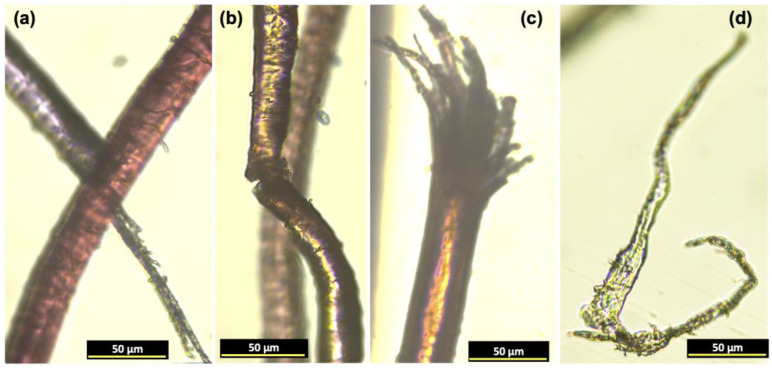
Damaged wool fibres: (**a**) a fibre torn along the length; (**b**) a neat cut in the transverse direction; (**c**) a shattered fibre, with protruding fibrils; (**d**) a ripped fragment.

The diameter of the reclaimed wool fibres ranged between ~15 and 50 µm, with the characteristic scales on their surface. The level of fibre damage is depicted in Appendix A, Figure A3. Also, as happened with cotton fibres, there are a lot of fibres where there are visible effects of the stresses imparted by the mechanical processes, thus contributing to an increased surface area.

#### 2.1.3. Identification by FTIR

Fibres with a peculiar morphology, like cotton or wool, can easily be identified with optical microscopy. But the most common man-made fibres are almost identical, with a circular cross-section and a plain surface appearance. In such cases, reflectance FTIR spectroscopy is the suitable technique for the identification of the functional groups through their bonding vibrations [38].

##### Cotton 

**Figure 2:**
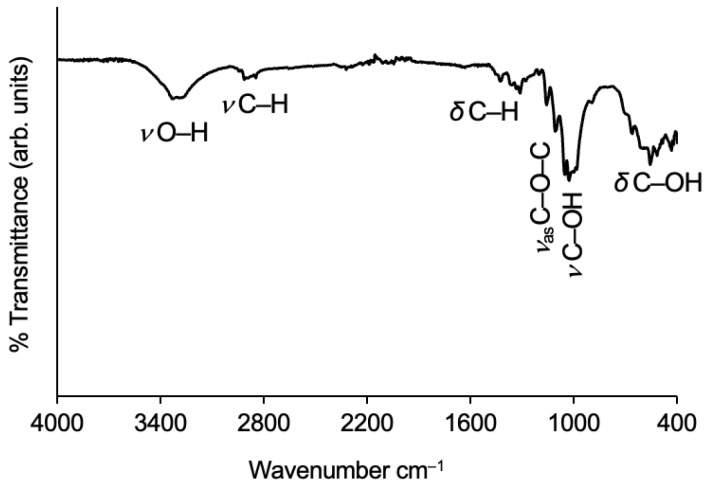
FTIR spectrum of the reclaimed cotton fibres used with the characteristic absorption bands identified.

The FTIR spectrum of reclaimed cotton fibres is sketched out in Figure 2. A characteristic spectroscopic band of cotton fibres reflects their hydrophilic nature, namely through the O–H stretching vibration. The strong and broad signal is caused by the large number of hydroxyl groups, leading to numerous hydrogen bonds [39], from which two adjacent peaks emanate at 3335 and 3285 cm^−1^, assigned to the intramolecular and intermolecular hydrogen bonds, respectively [40,41]. The band caused by the O–H absorbed water, which is normally visible around 1630 cm^−1^ in the deformation vibration mode [42], is absent due to the previous dehydration of the cotton fibres.

The C–H bond vibrations are also vital for cotton identification, which are present in the full length of the cotton spectrum. The broad band between 2940 and 2840 cm^−1^ refers to the C–H bond stretching vibration of the aliphatic methylene groups, an overlapping of both asymmetric (peak at 2916 cm^−1^) and symmetric (peak at 2849 cm^−1^) vibrations [40,41]. Regarding the C–H bonds’ in-plane bending vibration, the scissoring deformation is present around 1425 cm^−1^ [40,42,43], while the rocking deformation can be seen around 1315 cm^−1^ [42]. The FTIR bands for the CH2 out-of-plane deformation vibration are visible between 1337 cm^−1^ (wagging) and 1281 cm^−1^ (twisting) [44,45].

Evidence of the pyranose ring can be seen at the broad band below 1200 cm^−1^, through the asymmetric C–O–C bridge stretching around 1159 cm^−1^ and the anhydroglucose skeletal asymmetric stretching around 1109 cm^−1^ [39,40,42,46,47]. The stretching vibrations of C–OH bonds are represented at the bottom of the band, with peaks at 1053 and 1001 cm^−1^ for secondary and primary alcohols, respectively [40,42,45]. The out-of-plane vibrational mode of C–OH is visible around 557 cm^−1^ [42].

##### Polyester

**Figure 3:**
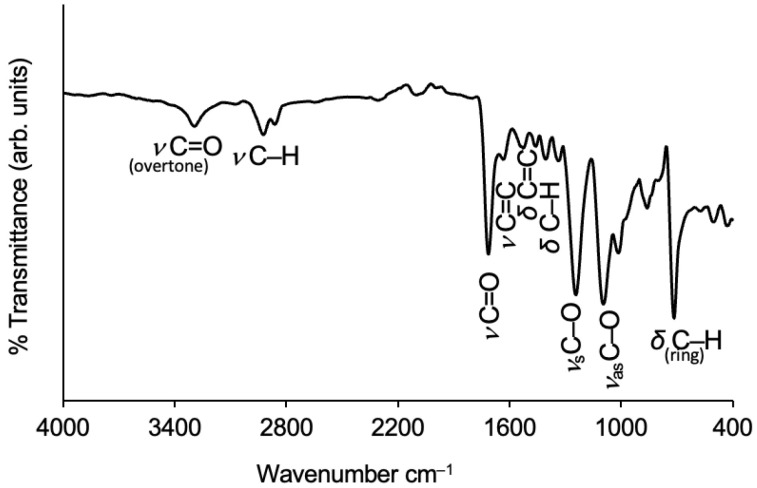
FTIR spectrum of the reclaimed polyester fibres used with the characteristic absorption bands identified.

The FTIR spectrum of reclaimed polyester fibres can be seen in Figure 3.

The emblematic FTIR band of polyester fibre is the C=O stretching vibration, with the peak being located near 1700 cm^−1^. In the spectrum of Figure 3, this band has a peak at 1713 cm^−1^. A fundamental vibration band may be accompanied by weak additional bands at multiple frequencies that are called overtones, and the weak band around 3300 cm^−1^ is the first overtone of the carbonyl stretching [40]. The two adjacent bands with peaks at 2923 and 2860 cm^−1^ refer to the C–H asymmetric and symmetric stretching of the aliphatic branch, respectively [40,48].

Regarding the presence of the benzene ring, the small bands with peaks at 1632, 1523 and 1458 cm^−1^ show some of those vibrational modes, namely, skeletal stretching of C=C, in-plane deformation of C=C and scissoring bending of C–H, respectively [40,49]. A band at a lower frequency (862 cm^−1^) is assigned to the C–H out-of-plane deformation vibration [49,50]. The last and strong band, below 800 cm^−1^, refers to the ring substitution, where C–H out-of-plane deformations occurred, ruled by the free hydrogen atoms which are adjacent to one another [50]. Two characteristic and strong bands of polyester fibres are assigned to the symmetric and asymmetric stretching vibrations of the C–O bond of the ester branch, with peaks at 1242 and 1094 cm^−1^, respectively [40,49]. These absorption bands are compatible with polyethylene terephthalate (PET), which is one of the most common polyester fibres in the textile industry. 

##### Wool

**Figure 4:**
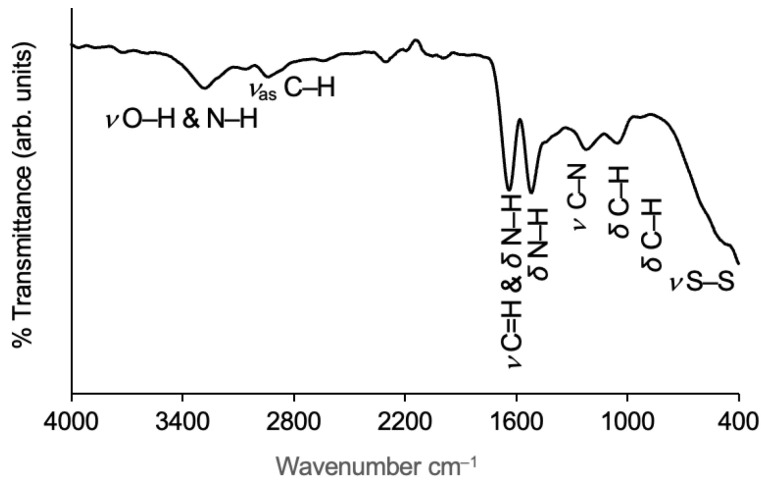
FTIR spectrum of the reclaimed wool fibres used with the characteristic absorption bands identified. The carbonyl band of the secondary amide in solid substances is a strong signal located between 1680 and 1630 cm^−1^ [40]. In this spectrum, the peak appears at 1639 cm^−1^. This band is mainly associated with the C=O stretching but also applies to the primary amine NH2 deformation [40,51]. Regarding the deformation vibration of secondary amides (N–H, solid materials), the band is located between 1570 and 1515 cm^−1^ [40,52]; it is visible here around 1518 cm^−1^, which mainly derives from the in-plane N–H bending mode [51]. Between 1310 and 1200 cm^−1^, there is a weak signal of mixed vibration, with a peak at 1227 cm^−1^, mainly assigned to the C–N stretching of the amide linkages [40,51,53]. The near weak band with peak at 1055 cm^−1^ refers to the C–H in-plane bending [40,52]. The very weak band in the lower spectrum region reveals the presence of a disulphide bond, a typical covalent bond of protein molecules, through the stretching vibrational mode [40], giving more evidence of the presence of wool fibres.

The main bands identified in the infrared spectrum of the reclaimed wool fibres are drawn in Figure 4. The broad band that appears between 3550 and 3120 cm^−1^, with a peak at 3281 cm^−1^, is assigned to the primary amide N–H stretching, within the range of 3320–3270 cm^−1^ [40], and the O–H stretching vibration is also visible in this spectrum region (despite the wool fibres being dehydrated before the analysis, there are intermolecular hydrogen bonds) [38,40,51,52]. The other prominent band in this spectrum region refers to the C–H asymmetric stretching (from CH2), with the peak at 2938 cm^−1^ [38,40,52].

### 2.2. Characterization of the Silica Aerogel Composites

The manufacturing processes of the cotton-reinforced composites was based on a recent published work [9]. Briefly, the silica system consisted of TEOS-isoBTES (tetraethyl orthosilicate-isobutyltriethoxysilane) system prepared under a two-step catalysed sol–gel method, according to the molar ratio: 

Si:EtOH:H_2_O:CH_3_COOH:NH_4_OH = 0.82/0.18:7.79:9.86:4.8 × 10^−2^:9.09 × 10^−2^.

On the other hand, the polyester- and wool-reinforced aerogel composites were thoroughly studied to find the optimal conditions for their development. The most relevant researched topics and their respective results are presented in Appendix B. 

#### 2.2.1. Cotton-Reinforced Composites

Our previous work [9] presented a silica aerogel composite reinforced with reclaimed cotton fibres with a remarkable performance for the sound absorption coefficient, *α*. However, despite the good results (*α* = 0.8), the total sound absorption coefficient, considering the sum of *α* at all frequencies, was around 30% lower than the cotton matrix itself, for the frequency range set at 100–2000 Hz. As such, the topic was further exploited. One of the new hypotheses consisted of trying to increase the sound absorption coefficient as a function of the composites’ cotton load. The reasoning was supported by a literature study, whose results pointed to a direct relationship between the increasing content of fibres and better performance of the composites in terms of the sound absorption coefficient [24]. The other hypothesis intended to link the degree of the fibres’ alignment to the insulation performance, either thermal or acoustic, due to the features of the reclaimed fibres.

- Aerogel composites: general presentation and key properties

**Figure 5:**
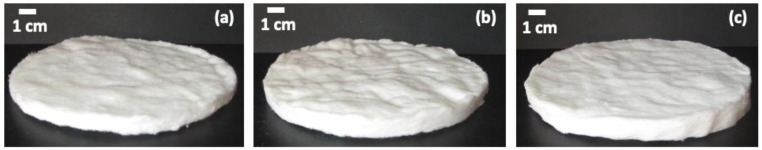
Scaled-up cotton-reinforced silica aerogel composites, custom-built for sound adsorption analyses: (**a**) conventional cotton mat with ~8.5 wt% of fibres; (**b**) conventional cotton mat with ~13.5 wt% of fibres; (**c**) compressed cotton mat with ~12.4 wt% of fibres.

Composites with two cotton fibre content values are presented in Figure 5a,b The version of the composite with a more aligned fibre mat is presented in Figure 5c.

An increased surface roughness from composite (a) to composite (b) is clearly perceived, due to the high fraction of cotton fibres, while from the composite (b) to the composite (c), there is again a decrease in surface roughness, a coherent outcome of the hot-pressing treatment of fibre layers.

The overall characterization and results can be seen in Table 1.

The low value of the Young’s modulus emphasizes the high flexibility of the composites. The relatively high standard deviation observed for composite (b), reinforced with the conventional mat with a high fraction of cotton fibres, can be a sign of a less homogeneous structure (also with an irregular surface), due to the high amount of fibres. In composite (c), made with the compressed mat, the Young’s modulus was reduced due to the evenness and increased flexibility imparted by the extra addition of fibres, when compared to the composite (a).

- Insulation Results: Thermal and Acoustic

The thermal conductivity results of composite (a) were poorer than expected, which can be explained by the high shrinkage experienced during drying that led to a high density, as disclosed in Table 1. By increasing the cotton content, in composite (b), the bulk density was reduced but the results on the thermal conductivity were further increased by ~20%. Despite the lower density, derived from the decreased shrinkage that was prevented by a higher amount of cotton fibres, the effective addition of solid matter worsened the insulation ability. Also, the high fraction of fibres diminished the cohesion of the silica matrix, causing the release of particles during the manufacture processes (which is the reason for the difference in the cotton wt% comparatively to the compressed cotton-reinforced dried composite, able to better accommodate the same mass of fibres). Regarding composite (c), the increased flatness imparted after the heat-pressing the cotton mat diminished the possibility of heat conduction through the raised ends of the cotton fibres within the blanket. Reclaimed fibres are shorter and curlier but tend to lay horizontally after being pressed, which is an advantage in terms of thermal conductivity, as conveyed by the obtained results, which showed a reduction from ~59 to ~51 mW m^−1^ K^−1^. This can be explained by the anisotropic values of cotton fibres, because the thermal conductivity is more than five times lower in the transverse direction [54].

**Figure 6:**
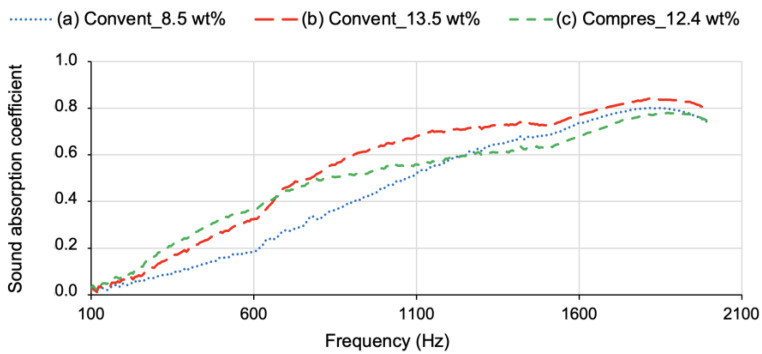
Sound absorption coefficient results for cotton–silica composites reinforced with conventional (a,b) and compressed (c) cotton blankets.

The sound absorption coefficient results are presented in Figure 6. The corresponding numeric values are already presented in Table 1, in terms of the sum of *α* at all frequencies of sound absorption coefficient results. The better performance was achieved for the composite with a high fraction of cotton fibres (conventional mat) and an uneven surface.

Regarding the results on the composites reinforced with the conventional reclaimed-cotton-fibre blankets, there is a direct correlation between acoustic performance and fibre content. The sound absorption coefficient was improved by more than 20%, derived from the fibres’ addition, when comparing the results of composite (b), with a fibre content of 13.5 wt%, with the ones of composite (a), with a cotton fraction of only 8.5 wt%. By increasing the amount of cotton fibres in the conventional mats, a higher number of gaps and air pockets are created [55], which led to an increased thickness (as can be seen in Table 1) and surface roughness, two beneficial features to enhance the sound absorption of the composites.

**Figure 7:**
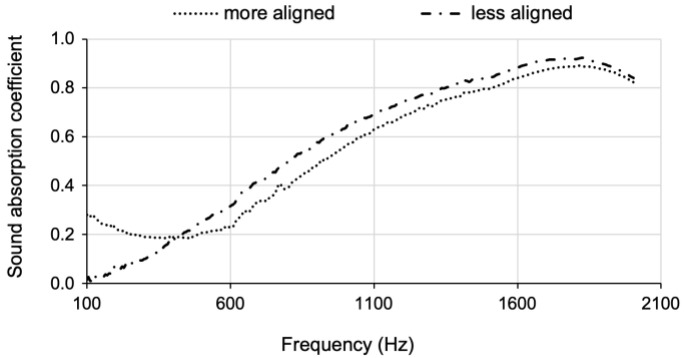
Sound absorption coefficient of two blankets made of reclaimed cotton fibres with different degrees of alignment.

On the other hand, the sound absorption coefficient decreased by ~10% from composite (b) to composite (c), the one reinforced with a conventional cotton blanket with 13.5 wt% of fibres and the other with a compressed cotton blanket, with 12.4 wt% of fibres, respectively. This not-so-good result in the acoustic performance of the composites is a consequence of the improved alignment of the cotton fibre layers, after the hot-pressing pre-treatment, despite the slightly larger thickness of composite (c), reinforced with 12.4 wt% of cotton fibres. In fact, composite (c) experienced a smaller shrinkage, due to the more regular reinforcement matrix, which also led to an increased flatness. However, the evenness of the composites proved to be detrimental in terms of the sound absorption coefficient. This conclusion was supported by the results of an extra evaluation carried out between two blankets made of reclaimed cotton fibres with different degrees of alignment: one roughly aligned in a manual carding (not so packed, with a bulk density of 59 kg m^−3^); the other one was an industrial, and thus more aligned, cotton blanket (*ρ* = 68 kg m^−3^). The thickness of both specimens was 9 mm, and the results are presented in Figure 7.

Similar evidence was described in another work that compared unprocessed raw jute fibres with carded fibres, where the evaluation of the sound absorption coefficient was accomplished with predicted values with two empirical models but also through experimental measurements. The less ordered sample of jute fibres presented better results compared to the carded sample [23]. Seemingly, the compressed layers of fibres tend to make the diffusion of the air disturbance difficult, reflecting the sound waves instead of absorbing them [56]. These findings are in line with the study of the EU [22], which says that lining up the natural fibres in mats “appears to slightly reduce the sound absorption”.

- Factors affecting thermal and acoustic insulation

**Figure 8:**
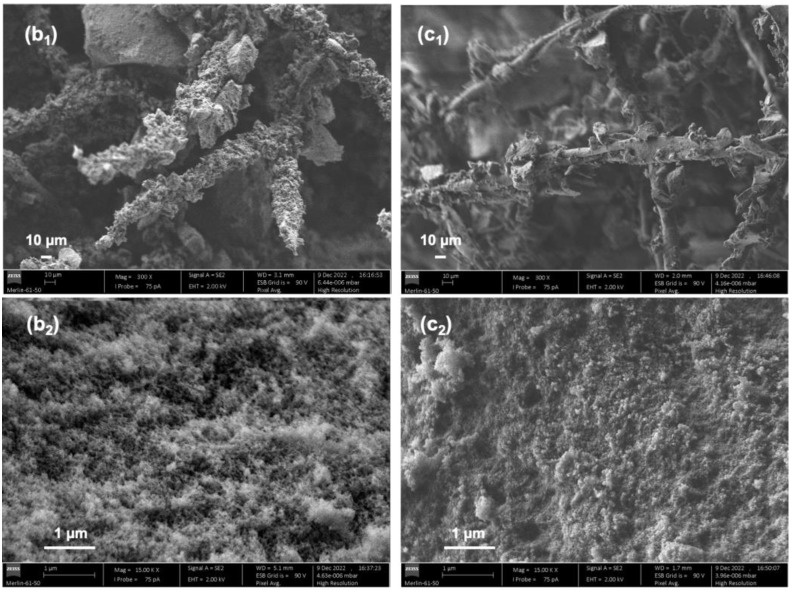
SEM images of the cotton–silica aerogel composites with a higher fraction of cotton fibres, where (**b**) refers to the conventional cotton mat with ~13.5 wt% of fibres and (**c**) refers to the compressed cotton mat with ~12.4 wt% of fibres. The subscripts (**1**) and (**2**) correspond to a 300× and 15,000× magnification, respectively.

The morphology of composites (b) and (c) was observed through SEM (Figure 8) to better understand the thermal and acoustic insulation performances.

Subtle differences can be perceived. In Figure 8b_1_, the cotton fibres are seen as if they were placed in two different planes, while in Figure 8c_1_ (compressed layers), the cotton fibres seem more levelled. Regarding the structure of the silica matrix (15,000× magnification), the composite made with the compressed layers of cotton (Figure 8c_2_) appears to have a more regular arrangement of the network, while the composite built with the conventional mat (Figure 8b_2_) seems more uneven. Also, at a first glance, the compressed-layer cotton composite seems to have a denser matrix, thus a reduced porosity.

It is known that surface roughness influences the acoustic ability of materials [17]. In terms of the sound absorption coefficient, the seemingly more disordered composite (b) exhibited the better performance. On the other hand, the more regular composite (c) performed better in terms of thermal insulation.

**Figure 9:**
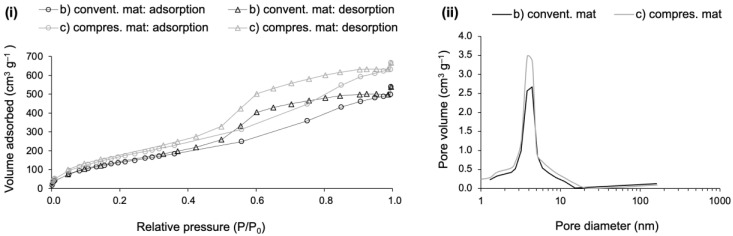
Sorption isotherms and BJH analysis of cotton–silica aerogel composites with a higher fraction of cotton fibres: (b) the composite reinforced with the conventional mat and (c) the composite reinforced with the compressed mat. Part (**i**) refers to the sorption isotherms (circles represent the adsorption branch; triangles represent the desorption branch) and part (**ii**) refers to the pore size distribution.

Composites (b) and (c) were also characterized for textural properties, in terms of specific surface area, pore dimensions and porosity. The porous structure of the composites was already unveiled by the SEM images, but mainly in terms of macroporosity. SEM imaging is a very useful resource to draw the big picture in terms of real pore dimensions, often being used to complement the evaluation carried out by BJH analysis through N_2_ sorption, which mainly considers the mesopore range [57]. Due to the presence of a hysteresis loop, the pore dimension assessment was performed at the desorption branch of the isotherm, which corresponds to a more stable thermodynamic condition of the adsorbate [58]. Additionally, the pore dimensions were also computed by another method that covers the whole range of the existing pores, because there are authors defending that the desorption branch of a type H2 hysteresis loop may give rise to misleading results [59]. All the obtained results can be seen in Figure 9 and Table 2.

The sorption curves of Figure 9i are type IV isotherms, with a hysteresis loop classified as type H2, according to IUPAC classification [59]. The initial part of the adsorption branches displays a curve with a steeper slope, which is linked to a moderate presence of micropores, as confirmed in the pore size distribution (Figure 9ii). As a side note, a vertical initial step indicates a massive presence of micropores [59,60]. The well-pronounced hysteresis relates to the mesoporosity and capillary condensation, in Figure 9i, while the loop shape of the isotherm branches is generally related to some pore restriction and a large pore size distribution [60,61]. Moreover, the mesopore size can be predicted as a function of the hysteresis position in terms of relative pressure, because the larger the p/p0, the larger the mesopore dimensions [60]. For both isotherms, the hysteresis is placed around the relative pressure of 0.6, which corresponds to a BJH average small mesopore diameter, being ~4 nm for both composites (see Figure 9i and Table 2). In terms of pore volume, it is slightly higher for the composite manufactured with compressed layers (see Figure 9ii). However, the limitations of the technique must be emphasized, as it does not reflect the real average pore dimensions in their full range. According to a recent IUPAC report, for pore sizes lower than 10 nm, the real dimensions will be underestimated by ~20–30% [61].

The porosity of composite (b) is slightly higher than composite (c) (see Table 2 and Figure 8), which can be explained by the lofty layers of the reclaimed cotton fibres used in composite (b). This resulted in slightly higher average pore dimensions (see *V*_pore_ and *D*_pore_), that in turn led to a smaller surface area (*A*_BET_) when compared to the composite (c). By observing the *V*_p,BJH_ and *D*_p,BJH_ results, the smaller pore dimensions reflect the restricted range measured by this method, which neglects most of the macropores. Nevertheless, after those results, it is possible to conclude about the existence of a high amount of macropores, due to the large difference in pore dimensions between the BJH method and the other measurement method that considers the pore dimensions in their full range.

Based on the results of *V*_pore_ and *D*_pore_, the better sound adsorption coefficient of composite (b) can be partly explained by the higher average pore dimension, while the better thermal insulation performance of composite (c) can be somehow explained by the smaller pore dimensions. These conclusions are in line with those of Begum and colleagues [62]. As already seen in Table 1, the compressed layers of fibres conferred quite different properties when compared to the conventional layers of reclaimed cotton fibres. The results of the analysis of the pore dimensions lead to similar conclusions.

As a final remark, the ideal conditions to produce silica aerogel insulation materials reinforced with cotton fibres will depend on the desired application, be it thermal or acoustic. If both functionalities are sought, a compromise must be engineered, weighted by the relevance of each property.

#### 2.2.2. Polyester-Reinforced Composites

As mentioned in Appendix B, the scaled-up polyester-reinforced silica composites were developed for the precursor system TEOS–VTMS (tetraethyl orthosilicate–vinyltrimethoxysilane) under the percentage molar ratios of 73-27 and 82-18. Additionally, two fractions of reclaimed polyester fibres were studied.

- Aerogel composites: general presentation and key properties

**Figure 10:**
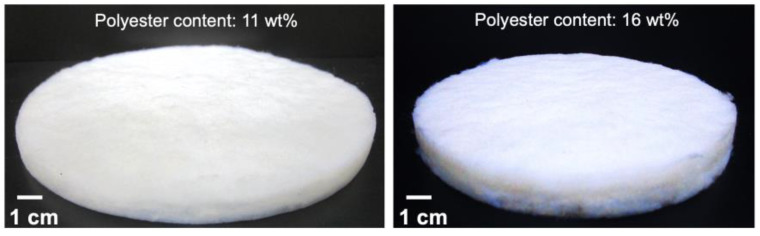
Polyester–silica aerogel composites with smaller and higher content of reclaimed fibres.

The appearance of the composites with both fractions of polyester fibres can be seen in Figure 10. The structural characterization and performance are detailed in Table 3. The polyester-reinforced composites had a reduced shrinkage when compared to the cotton-reinforced composites, as can be seen when comparing Table 3 vs. Table 1. 

Apart from the different co-precursor that was used here (VTMS in substitution of isoBTES), the other reason for the good results can be attributed to the higher amount of polyester fibres that were able to be accommodated in the same amount of the initial solution. The small differences in the final composites in terms of wt% of polyester fibres (by comparing the two different molar ratios for the equivalent mass of polyester) are related to the difference in moles of Si, around ~11% less for the composites developed under the molar ratio of 82-18. Because the volume of reactants was kept constant for all composites, this led to slightly less weighty final composites when compared to the ones developed under the molar ratio of 73-27; hence, the composites developed under the molar ratio of 82-18 contain a higher wt% of polyester fibres.

- The Young’s modulus of the polyester-reinforced composites is comparatively higher than that of the cotton-reinforced composites. A plausible reason might be that the smallest non-hydrolysable branch of the VTMS co-precursor, when compared to the longer branch of the isoBTES precursor, imparted flexibility to the silica–cotton composites (see Table 1). But the main reason seems to be the regularity of the polyester fibres, because the absence of cavities (like the cotton fibres) or a crimped character (like the wool fibres) does not favour the dissipation of the stress load [63]. According to Lu and colleagues, straight fibres “provide a stronger reinforcement to the aerogel composites, compared with the curved ones” [63]. Among the TEOS-VTMS systems of polyester–silica composites, by increasing the fraction of fibres, the composites gain in terms of flexibility, as denoted by the lower value of the Young’s modulus presented in Table 3. The significant difference in dimensions between the fibres’ diameter and pore/secondary particles’ dimension creates unbalanced responses to the received stress loads, which can explain the high standard deviation of some composites. Insulation results: thermal and acoustic

The low thermal conductivity of polyester fibres along with the low densities of the prepared composites led to the excellent results in terms of thermal conductivity, at the level of the values of the thermal conductivity of air. A slightly decreased performance in the composites with a high fraction of polyester was recorded, but they still displayed a very good result, despite the increased value of the standard deviation.

The weakness of these composites relies on the quite lower performance in terms of sound absorption, whose results are presented in Table 3, when compared to the cotton-reinforced composites, despite the generally increased thickness of the polyester-reinforced composites. These results are even poorer when compared to the polyester matrix itself. A mat of reclaimed polyester fibres, with a thickness of 14 mm and a density of 37 kg m^−3^, displayed an absorption coefficient sum as high as 171, around double the maximum value attained by the polyester–silica composites. Regarding the peak values of the sound absorption coefficient, it was 0.59 for the composite at the high fraction of polyester fibres, developed under the 82-18 molar ratio, while a peak of ~0.8 was accomplished for the mat of reclaimed polyester fibres.

**Figure 11:**
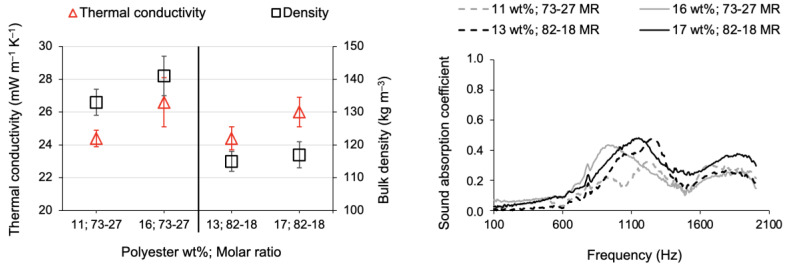
Overall insulation performance of reclaimed polyester fibre-reinforced silica aerogel composites, manufactured with two polyester fractions and two molar ratios (MR) of TEOS-VTMS.

The overall performance of the composites is summarized in Figure 11. The TEOS-VTMS polyester-reinforced composites developed under the molar ratio of 82-18 performed slightly better, both in thermal insulation and sound absorption coefficient.

- Factors affecting thermal and acoustic insulation

**Figure 12:**
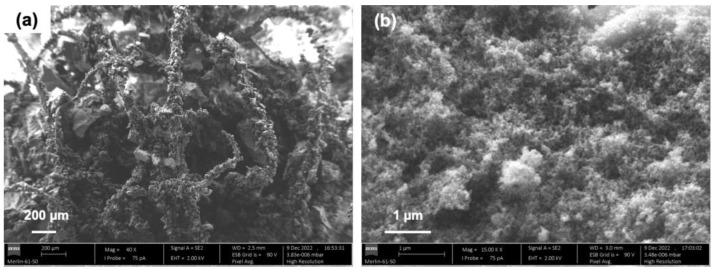
SEM images of the polyester–silica aerogel composites, prepared under a TEOS-VTMS molar ratio of 82-18, with 17 wt% of polyester fibres. Part (**a**) corresponds to 40× magnification, while part (**b**) corresponds to 15,000× magnification.

**Figure 13:**
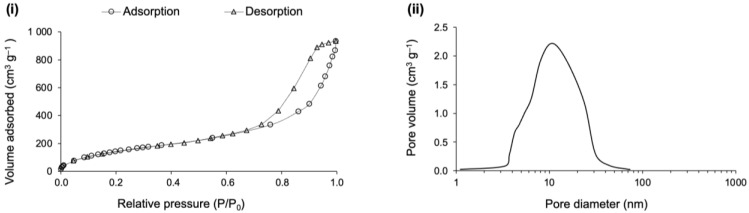
Sorption isotherms and BJH analysis of polyester-silica aerogel composites prepared under a TEOS-VTMS molar ratio of 82-18, with 17 wt% of polyester. Part (**i**) refers to the sorption isotherms (circles represent the adsorption branch; triangles represent the desorption branch) and part (**ii**) refers to the pore size and pore size distribution.

The morphology of the polyester-reinforced composite with the best performance in terms of the sound absorption coefficient was studied by SEM and nitrogen sorption isotherms to learn about the textural properties, trying to scrutinize the reasons for the lower acoustical ability when compared to the cotton-reinforced composites. The SEM analysis can be seen in Figure 12; the plots of the textural properties of the studied composite are presented in Figure 13, while the corresponding numerical data are presented in Table 4.

Regarding the SEM images, in Figure 12a, the polyester fibres are surrounded by the silica matrix, creating the presence of large macropores; Figure 12b reveals the porous network of the silica matrix, again with a large presence of macropores that are clearly visible. 

Regarding the sorption nitrogen measurements, the pore size of the polyester-reinforced composite displayed a quite different profile when compared to the cotton-reinforced composites. According to IUPAC classification [61], the isotherm layout is type IV, with a hysteresis loop of type H2b, which is plotted in Figure 13i. The H2b profile is an indication that the mesoporosity encompasses some pore blocking and also that the pore necks’ width has a wide size distribution [61], which is confirmed in Figure 13ii.

In the adsorption branch, the final saturation plateau (the Gurvich plateau), which is a common feature of isotherm IV, is missing here, an indication of the predominance of macropores [61]. The Gurvich plateau is observed when the pores are filled with the adsorbate, as the saturation is approaching, which generally occurs at relative pressures close to one, in the case of the nitrogen adsorbate, either for micro- or mesoporous materials. When the pores are too large, they will not be completely filled with the adsorbate at the saturation point, and consequently, an asymptotic increase in adsorption volume is observed [61]. The position of the hysteresis loop is another proof of the increased pore size of the polyester-reinforced composite, ranging from ~50 to ~150% larger, when compared to the cotton-reinforced composites. This is clear by comparing the plots of Figure 9 and Figure 13, because the larger the p/p0 for the hysteresis position, the larger the pores [49]. The numerical data render similar information, but other factors may have to be considered prior to concluding about the correlation between acoustic insulation performance and pore dimensions (as presented in Table 2 for the cotton-reinforced composites and in Table 4 for the polyester ones). For example, the systems of silica precursors and the reinforcement matrix properties seem to play a key role. Therefore, based solely on the pore dimensions, it is not straightforward to comprehend the performance of the polyester-reinforced composites in terms of the sound absorption coefficient if compared with the cotton-reinforced composites with smaller pore dimensions.

In terms of the literature results on polyester-based materials tested for the sound absorption coefficient, quite different results can be found. Two examples are presented here.

More than twenty years ago, a comprehensive study was carried out by Narang [64], who intended to grasp the potentialities of polyester fibres, aiming to tackle the possible adverse health effects imparted from insulation materials made of synthetic mineral fibres. Melt-binding nonwoven blankets of polyester fibres with quite different specificities were developed by studying a complete set of parameters and manufacturing conditions. A specimen made of hollow polyester fibres with a diameter of 26 µm, thickness of 65 mm and density of 7.7 kg m^−3^ (with 20 wt% of binding add-on, performed by low-melting-point conventional polyester fibres) displayed a sound absorption coefficient peak of ~0.64 [64] by considering the same interval of the measured frequencies as the present investigation work.

A recent work describes the in situ synthesis of silica aerogel in a polyester nonwoven needle-punching blanket (diameter of polyester fibres of ~29 µm) by using a two-step sol–gel process of TEOS, hydrophobized with tetramethylchlorosilane and ambient pressure drying (the fraction of fibres was not reported). A composite specimen with ~6.3 mm thickness, density of 210 kg m^−3^, porosity of 90% and mean pore diameter of 22 nm displayed a sound absorption coefficient peak of 0.44 at 2000 Hz (yet, better results were reported by the authors for higher frequencies that were not studied in the present research work). For the polyester blanket itself (with a porosity of 91% and density of 132 kg m^−3^), a smaller value of 0.24 was disclosed [20].

In view of those published works, the results of the present research are at a similar or even slightly better level of achievement. However, they are still below what was initially expected. Therefore, a question remains: what can be the reason for the not-so-good acoustic insulation performance?

Considering the increased thickness of the polyester-reinforced composite with better performance in terms of the sound absorption coefficient, more than ~50% when compared to the cotton-reinforced composite with the best result (namely 15.6 vs. 10.2 mm, Table 3 and Table 1, respectively), a reasonable explanation for the lower-than-expected results seems to rely on the intrinsic properties of the reinforcing fibres embedded within the aerogel, in this case the regularity of the polyester surface (a further proof for this reasoning is the sound absorption coefficient results of the wool-reinforced silica aerogel composites, shown below in this section, also manufactured with the TEOS-VTMS system).

This conclusion is supported by the literature, both in terms of fibre morphology and surface area. A study on the efficacy of round, trilobal and octolobal polyester fibres ascertained the better performance of octolobal fibres, due to the irregular profile and enlarged surface area. The same research study also analysed the effect of fibre diameter (~18 and ~33 µm), from which the thinner diameter performed better [65]. But this is not a consensus subject, because other authors suggest a direct relationship between the sound absorbing characteristics of the materials and the fibre diameter [21].

Concisely, the reclaimed cotton fibres, with uneven shape and increased surface area, seem to be the preferred choice for silica aerogel composites devised to perform as sound absorption barriers, while the reclaimed polyester fibres are the preferred option for the reinforcement of silica aerogel composites devoted to thermal insulation.

#### 2.2.3. Wool-Reinforced Composites

Similarly to the polyester–silica composites, the development of wool–silica composites also consisted of studying the effect of TEOS-VTMS molar ratio (see Appendix B) and the reinforcement fraction of the reclaimed wool fibres in the insulation ability of the materials.

- Aerogel composites: general presentation and key properties

**Figure 14:**
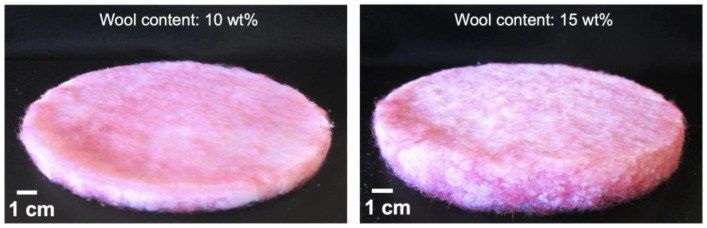
Wool–silica aerogel composites with studied fractions of wool fibres, manufactured under the TEOS-VTMS system.

Two of the wool–silica aerogels can be seen in Figure 14. The composites were prepared under the TEOS-VTMS percentage molar ratios of 73-27 and 82-18, where two fractions of fibres were used as the reinforcement matrices. The picture of the composites with the smaller content of wool (10 wt%) corresponds to the percentage molar ratio of 73-27, while the composite with the higher fraction of reclaimed fibres (15 wt%) corresponds to the percentage molar ratio of 82-18.

The overall features (structural, mechanical and insulation ability) are detailed in Table 5.

The lower values of the Young’s modulus of wool-reinforced composites compared to the polyester-reinforced composites (vide Table 5 vs. Table 3), despite the same precursor system, are mainly attributed to the airy and lofty trait of wool fibres that rendered the lowest density composites among the ones developed here. Contrarily to what was observed for the polyester-reinforced composites, the crimped and coiled features of wool fibres prevent the received stress loading from being fully effective, instead being dissipated around the fibres. This conclusion is supported by other studies [63]. Within the same system, the trends of increasing flexibility and reduced values of Young’s modulus were also observed here for the composites with higher fractions of wool fibres.

- Insulation results: thermal and acoustic

The wool fibres are intrinsically wavy and curly, thus allowing the presence of tiny air pockets that are responsible for their well-known superior thermal insulation. This trait is expressed by the very low thermal conductivity of the wool-reinforced silica aerogel composites presented in Table 5; on the other hand, the lack of flatness makes it difficult to compact the fibres into blankets to the same degree as the other fibres. Thus, the upper mass limit of wool to be used as reinforcement was comparatively lower than the polyester fibres, whose composites were made with the same precursor system (vide Table 5 vs. Table 3).

**Figure 15:**
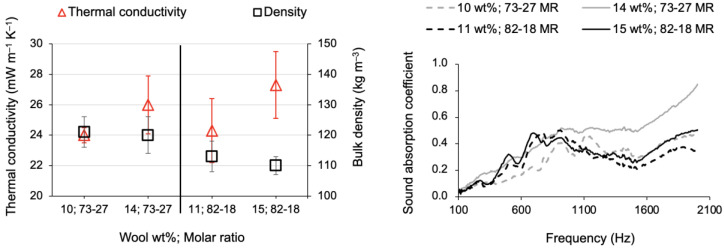
Overall insulation performance of reclaimed wool fibre-reinforced silica composites, manufactured with two fractions of wool and two molar ratios of TEOS-VTMS.

By comparing the insulation properties of the wool-reinforced composites, the TEOS-VTMS molar ratio of 73-27 performed slightly better compared to the 82-18 ratio, both in terms of thermal insulation and the sound absorption coefficient, as can be seen in Figure 15.

Around 2000 Hz, a maximum value of the sound absorption coefficient of 0.85 was recorded for the composite developed under the molar ratio of 73-27 and 14 wt% of wool fibres; moreover, the curve describes a rising trend, but the limit of that valid measurement is imposed by the equipment. The total sound absorption, by summing the obtained values at all frequencies, was around 20% more than the composite developed under the molar ratio of 82-18 and 15 wt% of wool fibres, the latter performing a maximum absorption of 0.5 at the frequencies ~700 Hz and above 1900 Hz.

- Factors affecting thermal and acoustic insulation

**Figure 16:**
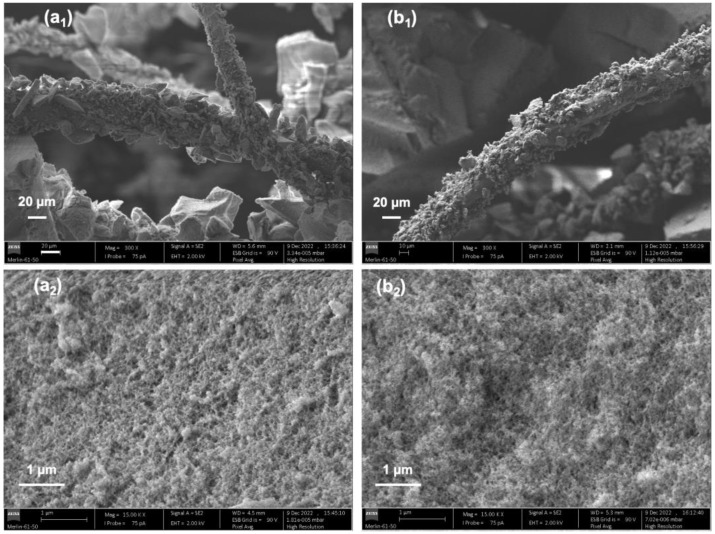
SEM images of the wool–silica aerogel composites with a higher fraction of wool fibres. Images (**a**) refer to the composite developed under the TEOS-VTMS molar ratio of 73-27, 14 wt% of wool fibres; part (**b**) refers to the composite developed under the TEOS-VTMS molar ratio of 82-18, 15 wt% of wool fibres. The subscripts (**1**) and (**2**) refer to 300× and 15,000× magnification, respectively.

The textural properties of the better performing composites in terms of the sound absorbing coefficient were observed under SEM analysis and characterized according BET and BJH techniques. The SEM images are presented in Figure 16.

The macroporosity of the silica aerogel composites is clearly visible in all pictures, particularly in Figure 1 and Figure 16b_1_. The silica network seems to display a more regular layout in Figure 16a_2_, which is in agreement with the previous findings of a longer gelation time with the 73-27 percentage molar ratio composites; Figure 16b_2_ shows an uneven surface, probably due to the abrupt formation of clusters caused by the shorter gelation time.

**Figure 17:**
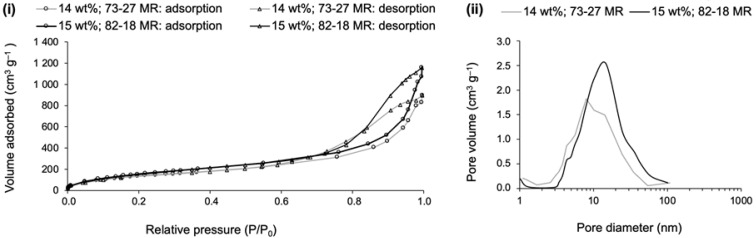
Sorption isotherms and BJH analysis for wool–silica aerogel composites prepared at different TEOS-VTMS molar ratios (MR), for the higher fraction of reinforcement (as described). Part (**i**) refers to the sorption isotherms (circles represent the adsorption branch; triangles represent the desorption branch) and part (**ii**) refers to the pore size and pore size distribution.

The textural results obtained after the nitrogen sorption analysis for the two best performing composites are presented in Figure 17.

For the wool-reinforced composites, the sorption isotherms displayed a slightly different profile when compared to the ones previously presented. According to the IUPAC criteria, they are also classified as type IV due to the presence of hysteresis (Figure 17i), in spite of the quasi-asymptotic final increase in the adsorption branch [57]. Therefore, the porosity of the composite can be related to a major macroporosity within a mesoporous vicinity. The desorption branch can be described by a type H2b hysteresis, a hint of mesoporosity with the same features of the polyester-reinforced composites, but a character of type H3 hysteresis is also visible, the sign of macropores not completely filled with N_2_ condensate [61]. The difference in the relative pressure of the closure of the desorption branch is related to the difference in the BJH pore dimensions [60]. As can be seen in Figure 17i, the closure of the composite with an 82-18 molar ratio happened at a higher relative pressure, thus presenting higher mesopore dimensions [60], as confirmed in Figure 17ii and in Table 6, showing the numerical data of the textural properties. The real average pore dimensions, that are calculated by including the macropores, have a slightly higher diameter for the composites developed under the 73-27 molar ratio.

The good thermal insulation of wool-reinforced aerogel composites can be understood considering the low densities and high porosities attained, along with the low thermal conductivity values of the wool fibres, which also include a diminished anisotropy value. The good performance in terms of the sound absorption coefficient is mainly attributed to the irregular surface of the wool fibres and with increased surface area due to the damage received during the harsh recovery processes. The better performance, with higher values recorded for the 73-27 molar ratio composite, is probably related to the higher average pore diameter (see Table 6).

#### 2.2.4. Final Remarks on the Reinforcement Matrices and Scaled-Up Composites

The compilation of the main results achieved is summarized here. Table 7 presents the overall characterization of the reinforcement blankets and the best performing composite in terms of the sound absorption coefficient for each type of reclaimed fibre.

**Figure 18:**
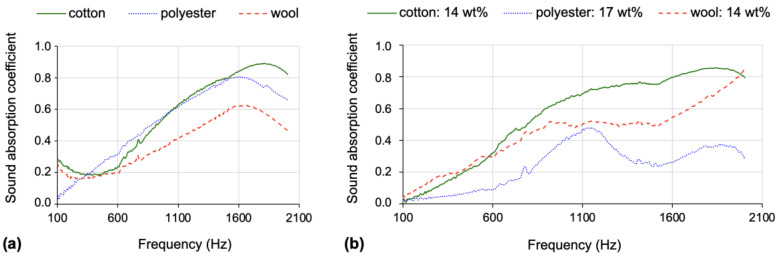
(**a**) Sound absorption coefficient of the fibre mats; (**b**) sound absorption coefficient of the silica aerogel composites reinforced with the expressed fraction of each fibre blanket (cotton, polyester, wool).

The initial premises regarding the ability of cotton fibres to absorb and attenuate sound waves were validated through this work, as illustrated in Figure 18, where part (a) refers to the reinforcement matrices and part (b) refers to the reinforced composites.

The cotton-reinforced composites manufactured with the TEOS-isoBTES precursor system, under the percentage molar ratio of 82-18, are undoubtedly the better sound absorption composite among the novel developments presented here (see Table 7 and Figure 18). To improve the not-so-good performance in terms of thermal insulation, the compression of the cotton layers is an expeditious solution to achieve a balance between thermal and acoustic insulation features.

The polyester-reinforced composites exhibited the worst performance in terms of the sound absorption coefficient, with the aerogel composites worsening the acoustic ability of the polyester mat (see Figure 18 and Table 7). On the other hand, optimal values of thermal conductivity were achieved for these composites (see Table 3 and Table 7). 

Despite the lower absolute mass of wool fibres that was used for the composite’s reinforcement, the results of the sound absorption coefficient were improved comparatively to the polyester–silica composites, by around twice or more, as can be seen in Table 3, Table 5 and Table 7. In fact, the wool–silica composites performed better than the wool matrix itself at most frequencies, with the global performance increased by ~15% (see Table 7). The thermal insulation results were also very good.

Concisely, the reclaimed cotton fibres, with uneven shape and increased surface area, seem to be the preferred choice for sound absorption silica aerogel composites, while the reclaimed polyester fibres are the preferred option for the reinforcement matrix of silica aerogel composites devoted to thermal insulation. Wool-reinforced composite aerogel was found to be the perfect option for thermal and acoustic insulation barriers simultaneously.

Currently, the main insulation materials consist of panels made from man-made mineral fibres and polymer open-cell foams, whose efficacy tends to prevail over the human health deleterious effects and ecological deficiency, respectively. Recent epidemiology studies on occupational exposure to man-made mineral fibres reported negative effects in the respiratory system [66], while climate urgency advocates for the oil-derived products to be replaced for environmentally favourable materials. The perfect material would be engineered to perform thermal and acoustic insulation, manufactured under the best production practices and using recovered materials. The silica aerogel composites presented here are intended to pave that way.

## 3. Conclusions

The global aerogel-based materials market is expected to grow at a high pace, driven by the increasing need for efficient thermal insulation materials to foster energy savings. 

Thermal and acoustic insulation abilities of silica aerogels reinforced with reclaimed textile fibres were presented here. Reclaimed cotton, polyester and wool fibres obtained from textile industry leftovers were used for the first time as the reinforcement matrices for silica aerogel composites, manufactured with feasible techniques, thus foreseeing a potential scale-up and future commercialization. The manufacturing of the composites was preceded by thorough optimization of the conditions, due to the need to scale up the samples for sound absorption measurements. TEOS was used as the main precursor, and isoBTES and VTMS were used as co-precursors, according to the type of fibre.

The cotton–silica aerogel composites were TEOS-isoBTES-based, with excellent results in terms of the sound absorption coefficient when 14 wt% of cotton fibres were used, presenting a peak value of *α* as high as 0.86. This excellent result was attributed to the irregularity and high surface area of the cotton fibres, further increased after the severe mechanical processes that turned the textile leftovers into reclaimed cotton fibres. However, the thermal insulation features of the aerogel composites became deteriorated, due to the high fraction of cotton, a very good thermally conductive fibre. Additionally, cotton fibres have high anisotropy, a detrimental feature further evidenced by the predominance of small and curly fibres, which tend towards misalignment. But a compromise was achieved between thermal and acoustic insulation, which consisted in hot-pressing the layers of reclaimed cotton fibres, thus making a more levelled and regular blanket. Due to that expeditious treatment, the thermal conductivity was reduced from ~59 to ~51 mW m^−1^ K^−1^, while the sound absorption coefficient decreased slightly. Yet, a very good acoustic insulation performance was achieved.

The low thermal conductivity of polyester-reinforced composites manufactured with the TEOS-VTMS system led to excellent results, at the level of the values of the thermal conductivity of air (24 mW m^−1^ K^−1^). However, the sound absorption coefficient was not so good, which was attributed to the regular and plane surface of the round polyester fibres. Due to the lower cost and easy availability of reclaimed polyester fibres, this would be the ideal reinforcement matrix for thermal insulation barriers.

The better results in both thermal and acoustic insulation were achieved by the wool-reinforced composites. The thermal conductivity values were less than 27 mW m^−1^ K^−1^, with most of the composites reaching the excellent result of ~24 mW m^−1^ K^−1^. In terms of the sound absorption coefficient, it achieved a peak value of 0.85 at ~2 kHz, a better performance than the wool matrix itself.

As a final remark, the cotton–silica aerogel composites are the better option for acoustic insulation barriers, while the polyester–silica aerogel composites are the better option for thermal insulation barriers. By comparing the results of wool–silica composites with those of polyester composites, there is no doubt on the appropriateness of the wool fibres for their acoustic-absorbing properties in tandem with thermal insulation. Notwithstanding all the findings in this pioneering work, further research is needed to broadly conclude about other available reclaimed fibres and test other alternative silica sources.

Apart from the proven efficacy of the developed silica aerogel composites, the use of reclaimed fibres is an ingenious way to tackle the inherent fragility of silica aerogels while promoting a circular economy.

## 4. Materials and Methods

### 4.1. Materials

Tetraethyl orthosilicate (TEOS, 98%) and vinyltrimethoxysilane (VTMS, 98%) were acquired from *Acros Organics;* ethanol (EtOH, ≥99.8%), ethyl acetate (EtOAc ≥ 99.8%) and heptane (99%) were bought from *Fisher Chemical*, while acetic acid (glacial, analytical reagent grade, used as the acid catalyst) was purchased from *Fisher Scientific*. *Acros Organics*, *Fisher Chemical*, and *Fisher Scientific* are all belonging to the *Thermo Fisher Scientific* group (Waltham, MA, USA). Hexamethyldisilazane (HMDZ, 98.5%) was supplied from *abcr GmbH* (Karlsruhe, Germany), 1 M ammonium hydroxide (puriss. p.a., used as the basic catalyst) from *PanReac AppliChem* (*ITW Reagents*, Barcelona, Spain). Isobutyltriethoxysilane (isoBTES, ≥95%) was purchased from *Aldrich Chemistry* and vinyltriethoxysilane (VTES, ≥98%) from *Aldrich*, the two brands of *Sigma-Aldrich* being members of the *Merck KGaA* (Darmstadt, Germany). The chemical reagents were used without further purification. The water for hydrolysis of precursors and for the aqueous solution of the basic catalyst was purified through the ultrapure water system *Milli-Q^®^* (Burlington, MA, USA). Reclaimed fibres of cotton and polyester were kindly provided in bundles by *SASIA* (Ribeirão, Portugal), and wool reclaimed fibres were kindly provided by *J. Gomes Lda.* (Covilhã, Portugal). Commercial hydrophilic cotton blankets, made of reclaimed fibres and zig-zag packed, were purchased from *Modelo Continente Hipermercados, S.A.*, (*Hidrofer*, Vila Nova de Famalicão, Portugal). 

### 4.2. Preparation of the Composites

**Figure 19:**
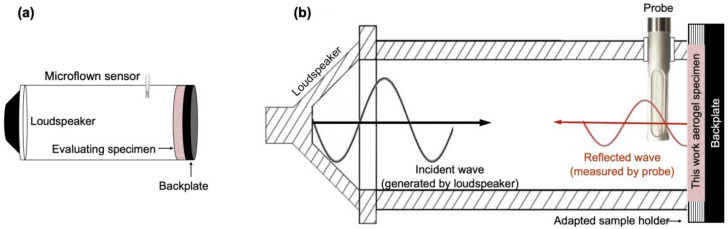
General settings of the impedance tube, where part (**a**) depicts the general measurement procedure and (**b**) sketches out the adapted procedure used in this work (part (**b**) was reproduced and adapted from [69], with permission).

TEOS was chosen as the main precursor because is the most affordable among the silanes [14,67] and considering its features of controllable hydrolysis and condensation rates [68]. The dimensions of the composites were adjusted to the requirements of the sound absorption apparatus, because its inner diameter is 10 cm and the specimen must be placed inside it, as sketched out in Figure 19a. However, it is quite difficult to obtain specimens of silica aerogel composites in those perfect dimensions, due to the inconstant value of shrinkage during the manufacturing processes. It is also a hard task to cut them from a higher dimension sample, because of the high porosity of these materials and due to the embedded fibres, which cause yet more difficulties in maintaining the regularity and integrity of the composites at the cut area. Also, in terms of result accuracy, errors can emerge due to any circumferential air gap between the specimen and the internal wall of the impedance tube [23]. All these questions were solved by changing the specimen dimensions and testing position. For that, an adaptable sample holder was placed between the entrance of the impedance tube and the backplate, where the silica aerogel composites with a diameter of ~12 cm were settled, as in Figure 19b.

#### 4.2.1. Cotton-Reinforced Composites

The cotton–silica aerogel composites were manufactured by following the previous procedure in terms of optimized stoichiometry [9]. Briefly, a two-step catalysed sol–gel method was followed. The precursor and co-precursor, i.e., TEOS and isoBTES, in a volume ratio of 4:1, were firstly mixed and hydrolysed for 24 h at 27 °C in a solution of ethanol, water and acetic acid. Then, the condensation reactions were trigged by the addition of 1 M ammonium hydroxide aqueous solution, under stirring, to form a sol and finally a gel (already in the presence of the fibres). The percentage molar ratios were TEOS/isoBTES:EtOH:H_2_O:CH_3_COOH:NH_4_OH = 0.82/0.18:7.79:9.86:4.8 × 10^−2^:9.09 × 10^−2^, in which the purified water of the 1 M ammonium hydroxide aqueous solution (from ammonia solution ~25% NH₃ in water) is included, along with the water needed for the hydrolysis of precursors. Moreover, to better control the amount of water added to the initial mixture of reactants, the acid catalyst (H_3_COOH) was used as received, in the concentrated state. 

The cotton carded blanket was cut with the same diameter of the Petri dish mould for the synthesis. After that, the layers of reclaimed cotton fibres were dehydrated using a laboratory oven thermostatically controlled at a temperature of 105 ± 2 °C, with ventilation, for ~4 h [70]. This is a mandatory procedure to promote the reproducibility of the experiments, due to the hydrophilic character of cotton fibres, with a moisture regain of ~8 wt%, when in a standard atmosphere of 65% relative humidity and 20 °C [71]. 

The pre-determined mass of fibres was then disposed layer-by-layer, over which the sol was poured. After gelation, the Petri dish was covered and made airtight to perform ageing at 50 °C for 3 days.

As these were scaled-up samples, a few adjustments were made after the gelation step to fit the demand of larger samples, because the diffusional time for the same effectiveness of silylation and solvent exchange is higher for larger gel sizes [72]. The surface modification was extended by 6 h (for ~54 h) and the silylating solution was exchanged at approximately the middle time. Moreover, an additional solvent exchange/washing was performed: the first one for 12 h at 40 °C and the second and the third at 50 °C, each one for 5 h. The aerogel composites were dried at 140 °C for 2 h 30 min. The study encompassed two wt% fractions of reclaimed cotton fibres, composed of orthogonally oriented individual layers. Due to the lack of flatness on most of the fibres, another set of experiments was also performed by compressing those individual layers of fibres (1 ton-f, at 105 °C, for 60 s). 

#### 4.2.2. Polyester-Reinforced Composites

The fibre layers were directly used from the polyester carded blanket, without dehydration, due to the hydrophobic nature of the polyester fibres [73]. Apart from that, the polyester–silica aerogel composites were prepared by following the same procedures of the cotton-reinforced composites. However, the co-precursor and the post-synthesis solvent had to be changed, from isoBTES to VTMS and from EtOAc to heptane, respectively, to enhance the compatibility with the different reinforcement fibres. Further details on the experimental work and procedure optimization are given in Appendix B. 

The composites were developed under two molar ratios of silica precursor to co-precursor, one as TEOS-VTMS = 82-18 (equal to the one applied for TEOS-isoBTES) and another one as TEOS-VTMS = 73-27.

#### 4.2.3. Wool-Reinforced Composites

The wool–silica aerogel composites were developed with the TEOS/VTMS precursor/co-precursor system, under the same molar ratios that were followed for the polyester–silica aerogel composites mentioned before, also after it had been realized that the TEOS/isoBTES precursor/co-precursor system led to unsatisfactory results. Likewise, the post-synthesis treatments were carried out with heptane. Wool textile fibres are hydrophilic, with a moisture regain of ~14–18% [74]; thus, its dehydration was carried out, as in the cotton-reinforced composites, aiming to promote the reproducibility of the experiments. Similarly to the polyester-reinforced composites, two fractions of wool were used, the lower amount envisaging to create a thermal barrier and the higher amount with the purpose to create a sound-absorbing material. 

The relevant experimental work and the corresponding results to the optimized manufacturing conditions are described in Appendix B.

#### 4.2.4. Characterization Techniques

The bulk density of the reinforced composites was assessed by weighing regular specimens in a microbalance (10^−5^ g precision) and measuring them with a calliper (0.01 mm resolution). The morphology of the reclaimed fibres was analysed by a fluorescence microscope *LEICA DM 5000B* (*Leica Microsystems*) at 40× magnification. Thermal conductivity of the composites was assessed by the transient plane source method, with a *Thermal Constants Analyzer TPS 2500 S (Hot Disk)*, at 20 °C. The chemical identification of reclaimed fibres by FTIR was studied with an *IRAffinity-1S*, using a single-reflection diamond ATR accessory (*Specac*). Mechanical properties of the composites were evaluated through the uniaxial compression at 1 mm s^−1^, in an *Inspekt mini-series* machine (*Hegewald & Peschke*). The microstructure of the composites was observed through an FE-SEM (Field Emission Scanning Electron Microscope) equipped with a *Gemini II* column (*Zeiss, Merlin*). The sound absorption coefficient (*α*) of the composites was determined by using a custom-built impedance tube equipped with a *Microflown Technologies* system coupled to a *PU* probe (sound pressure (*P*) and particle velocity (*U*)). The pore dimensions and specific surface areas were measured by nitrogen adsorption–desorption isotherms at 77 K using an *ASAP 2000* (*Micromeritics Instrument Corporation*). The specific surface area was measured by the Brunauer–Emmett–Teller (BET) method, derived from the region of the adsorption isotherm that corresponds to the assumption that the adsorbate covers the external surface and the pore walls with a single layer of atoms [58]. The specific pore volume and average pore diameter were estimated according to the Barrett–Joyner–Halenda (BJH) method applied to the desorption isotherm at relative pressures > 0.35 [57]. Due to the limitations of the BJH technique, that neglects the micro- and most of macropores [57,61], the total pore volume and average pore size were also computed according to Equations (1) and (2), defended as a better approximation for the actual pore size reality [75]. $V_{\mathrm{pore}}$ is calculated from the aerogel bulk density and aerogel skeletal density; $D_{\mathrm{pore}}\;$ is then obtained with $V_{\mathrm{pore}}$ and using the result of the surface area computed by BET analysis.

(1) $$V_{\mathrm{pore}}=\frac{1}{\rho_{\mathrm{bulk}}}-\frac{1}{\rho_{s}}$$

(2) $$D_{\mathrm{pore}}=\frac{4V_{\mathrm{pore}}}{A_{\mathrm{BET}}}$$

The porosity was computed according to Equation (3) by considering the previously determined bulk density ($\rho_{b}$) and the measurement of skeletal density ($\rho_{s}$), evaluated by a helium pycnometer *AccuPyc 1330* (*Micromeritics Instrument Corporation*).

(3) $$\mathrm{Porosity}\;\left(\%\right)=\left(1-\frac{\rho_{b}}{\rho_{s}}\right)\times 100$$

Prior to the above analyses, both for helium pycnometry and N_2_ sorption isotherms, the specimens were degassed under a partial vacuum (~800 mbar) at 80 °C for 60 h in order to remove residual gases, moisture and other impurities [57].

## Figures and Tables

**Table 1:** Structural and mechanical characterization and insulation performance of the scaled-up silica aerogel composites reinforced with reclaimed cotton fibres (TEOS-isoBTES system).

Composites	Cotton		Shrinkage (%)		Thickness (mm)	Density (kg m^−3^)	*E* (kPa)	*k* (mW m^−1^ K^−1^)	*a* (∑ 100–2000 Hz)
	(g)	(wt%)	Diameter	Volume					
(a) convent. mat	2.4	8.5	8.9 ± 0.7	52.3 ± 1.5	8.2 ± 0.3	214 ± 9	64 ± 4	49.7 ± 0.3	146
(b) convent. mat	4.1	13.5	4.2 ± 0.5	43.6 ± 0.8	10.2 ± 0.4	197 ± 3	65 ± 17	59.2 ± 0.3	181
(c) comp. mat	4.1	12.4	3.3 ± 0.1	41.1 ± 2.1	10.4 ± 0.2	204 ± 5	58 ± 4	51.1 ± 0.2	167

**Table 2:** Textural properties of cotton–silica aerogel composites prepared under TEOS-isoBTES percentage molar ratio of 82-18.

Composites	Skeletal Density (kg m^−3^)	Porosity (%)	*A*_BET_ (m_2_ g^−1^)	*V*_p, BJH_ (cm_3_ g^−1^)	*D*_p, BJH_ (nm)	*V*_pore_ (cm_3_ g^−1^)	*D*_pore_ (nm)
(b) convent. mat	1319 ± 20	85.3 ± 0.3	555.5 ± 4.6	0.82 ± 0.02	3.8 ± 1.1	4.33 ± 0.07	31.2 ± 0.5
(c) comp. mat	1227 ± 13	83.4 ± 0.4	691.9 ± 6.7	1.04 ± 0.02	3.5 ± 1.2	4.09 ± 0.10	23.6 ± 0.6

**Table 3:** Structural and mechanical characterization and insulation performance of the scaled-up silica aerogel composites reinforced with reclaimed polyester fibres (TEOS-VTMS system).

Composites	Polyester		Shrinkage (%)		Thickness (mm)	Density (kg m^−3^)	*E* (kPa)	*k* (mW m^−1^ K^−1^)	*α* (∑ 100–2000 Hz)
	(g)	(wt%)	Diameter	Volume					
73-27 molar ratio %	3.5	10.9	2.4 ± 0.3	18.5 ± 0.5	9.6 ± 0.2	133 ± 4	245 ± 30	24.4 ± 0.5	62
	5.1	16.0	3.5 ± 0.0	16.4 ± 1.0	14.8 ± 0.5	141 ± 6	215 ± 20	26.6 ± 1.5	73
82-18 molar ratio %	3.5	13.0	1.4 ± 0.1	10.6 ± 0.1	14.9 ± 0.1	115 ± 3	247 ± 15	24.4 ± 0.5	64
	5.0	17.1	2.5 ± 0.2	10.2 ± 0.4	15.6 ± 0.4	117 ± 4	174 ± 5	26.0 ± 0.9	71

**Table 4:** Textural properties of polyester–silica aerogel composites prepared under an 82-18 molar ratio, with 17 wt% of polyester.

Composites	Skeletal Density (kg m^−3^)	Porosity (%)	*A*_BET_ (m^2^ g^−1^)	*V*_p, BJH_ (cm_3_ g^−1^)	*D*_p, BJH_ (nm)	*V*_pore_ (cm_3_ g^−1^)	*D*_pore_ (nm)
82-18 molar ratio %; Polyester 17 wt%	1119 ± 87	89.5 ± 0.8	577.2 ± 5.4	1.45 ± 0.05	9.3 ± 0.8	7.66 ± 0.26	53.1 ± 1.6

**Table 5:** Structural and mechanical characterization and insulation performance of the scaled-up silica aerogel composites reinforced with reclaimed wool fibres (TEOS-VTMS system).

Composites	Wool		Shrinkage (%)		Thickness (mm)	Density (kg m^−3^)	*E* (kPa)	*k* (mW m^−1^ K^−1^)	*α* (∑ 100–2000 Hz)
	(g)	(wt%)	Diameter	Volume					
73-27 molar ratio %	2.7	10.2	0.3 ± 0.2	10.0 ± 3.9	13.1 ± 0.5	121 ± 5	91 ± 11	24.0 ± 0.5	94
	3.3	13.6	2.4 ± 0.5	11.4 ± 1.8	13.4 ± 0.4	120 ± 6	83 ± 7	26.0 ± 1.9	144
82-18 molar ratio %	2.6	11.3	0.2 ± 0.2	5.7 ± 3.5	13.5 ± 0.5	113 ± 5	65 ± 5	24.3 ± 3.1	92
	3.6	14.8	0.1 ± 0.1	2.9 ± 1.0	13.8 ± 0.2	110 ± 3	56 ± 10	27.3 ± 2.2	104

**Table 6:** Textural properties of wool–silica aerogel composites prepared at different TEOS-VTMS molar ratios, at higher fractions of fibre reinforcement.

Composites	Skeletal Density (kg m^−3^)	Porosity (%)	*A*_BET_ (m_2_ g^−1^)	*V*_p, BJH_ (cm_3_ g^−1^)	*D*_p, BJH_ (nm)	*V*_pore_ (cm_3_ g^−1^)	*D*_pore_ (nm)
73-27 molar ratio %; Wool 14 wt%	1128 ± 40	89.4 ± 0.6	531.4 ± 5.9	1.39 ± 0.05	7.57 ± 1.45	7.46 ± 2.43	56.15 ± 2.43
82-18 molar ratio %; Wool 15 wt%	1062 ± 13	89.6 ± 0.3	619.7 ± 6.2	1.80 ± 0.06	10.01 ± 1.03	8.15 ± 0.22	52.64 ± 1.29

**Table 7:** Characterization of the fibre reinforcement blankets and the corresponding sound absorption coefficient, on the left side; on the right side of the table, the results of the best performing silica aerogel composites on each type of reclaimed fibre, both in terms of thermal conductivity and the sound absorption coefficient.

	Reinforcement Matrices				Best Performing Aerogel Composites		
Fibres	Thickness (mm)	Density (kg m^−3^)	*α* (100–2000 Hz)		*k* (mW m^−1^ K^−1^)	*α* (100–2000 Hz)	
			Sum	Peak		Sum	Peak
Cotton	9	68	180	0.89	59.2	179	0.86
Polyester	14	37	171	0.81	24.4	64	0.53
Wool	13	42	126	0.63	26.0	144	0.85

**Figure A4:**
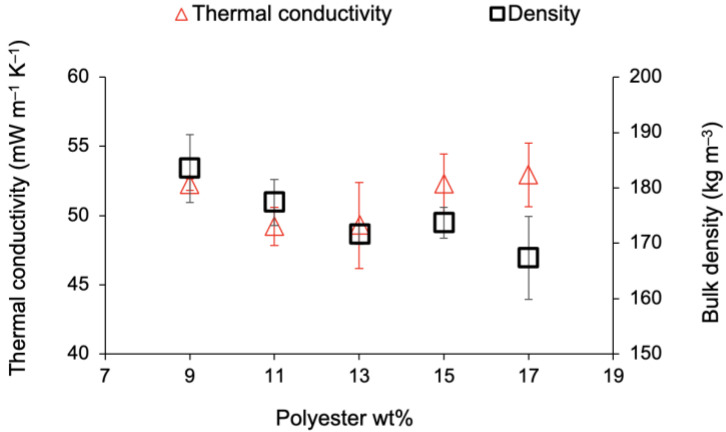
Influence of fibre amount on the thermal conductivity and density of polyester-reinforced composites (TEOS-isoBTES system).

**Figure A5:**
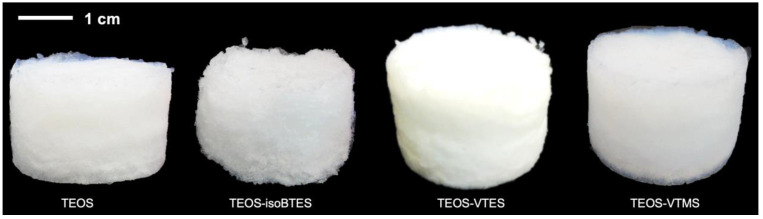
Appearance of the polyester-reinforced aerogel composites, developed with different co-precursors (the silica system used is described under each image), with the same mass of polyester fibres, and keeping the volume of the precursor and co-precursor constant.

**Figure A6:**
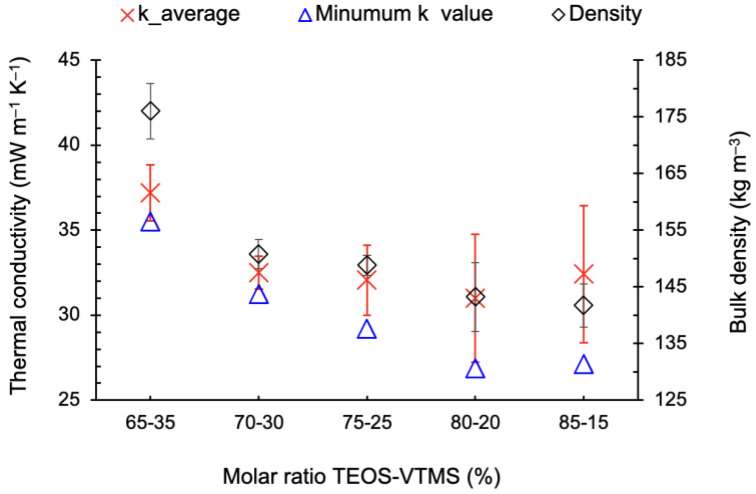
Results of the thermal conductivity and bulk density of the studied TEOS-VTMS percentage molar ratios in the polyester-reinforced aerogels.

**Figure A7:**
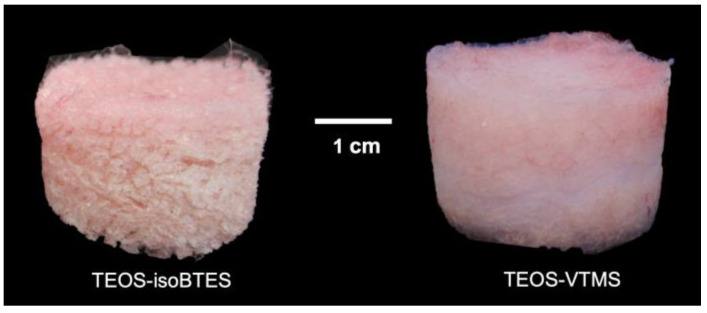
Wool–silica aerogel composites aspect, developed with different precursor systems, as indicated.

**Figure A8:**
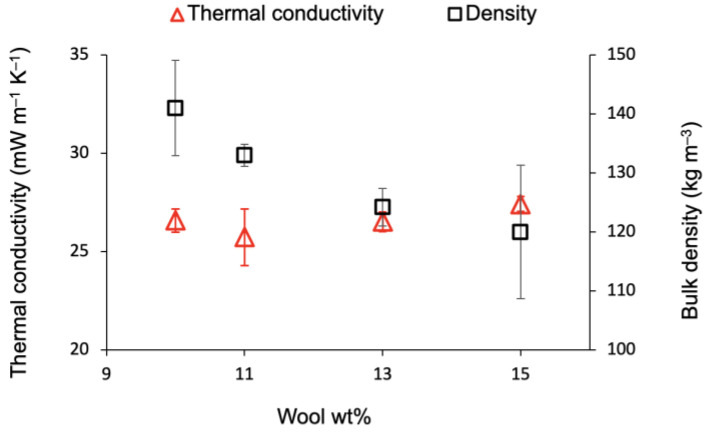
Influence of fibre amount on the thermal conductivity and bulk density of wool-reinforced composites (TEOS-VTMS system).

## Data Availability

Data will be available upon request to the authors.

## Appendix B. Study of the Optimal Processing Conditions

The development of the wool- and polyester-reinforced composites started under the format of small cylindrical samples (diameter ~3 cm), offering a simpler development protocol to reach the optimal conditions for the composites’ manufacturing.

1. Polyester-Reinforced Silica Aerogel Composites

The first synthesis consisted of TEOS-isoBTES as the silica precursor (82-18 percentage molar ratio) and EtOAc as the washing solvent. But that composition/conditions did not work well for silica–polyester composites, because the density of the materials obtained was higher than expected (>230 kg m^−3^), and they also had high values of thermal conductivity (>46 mW m^−1^ K^−1^). Therefore, several studies were carried out to find out the best processing conditions and chemicals, and the most relevant are detailed below.

- Post-Synthesis Solvent

The use of EtOAc was intended to promote a safer production route [76], but after the first synthesis with polyester fibres, it became clear that, in this case, it was not the best solvent. The lower polarity of polyester when compared to the cotton fibres [77] might be the reason for the non-formation of the hydrogen bonds that seemed to have occurred in the cotton-reinforced composites. In fact, EtOAc is an aprotic polar solvent with the ability to act as a hydrogen bonding acceptor [78], while the hydroxyl groups of cellulose have the ability to act as hydrogen bonding donors [79]. Polyester does not have these hydroxyl groups. Consequently, two non-polar solvents were used for comparison, namely, n-hexane, which according to the literature is regularly used in the post-processing of gels reinforced with polyester fibres [80,81], and n-heptane, a less hazardous option, due to the known high toxicity of hexane [76]. The preliminary results can be seen in Table A1, with heptane as the post-synthesis solvent providing the better results. The bulk density of the composites was lowered by ~30 and ~6% when compared to the EtOAc and hexane solvents, respectively, and also with the lower values in thermal conductivity.

**Table A1:** Structural and insulation performance of the scaled-up silica aerogel composites reinforced with reclaimed polyester fibres (TEOS-isoBTES system).

Post-Synthesis Solvent	Polyester		Shrinkage (%)			Density (kg m^−3^)	*k* (mW m^−1^ K^−1^)
	(g)	(wt%)	Diameter	Height	Volume		
EtOAc	0.16 ± 0.0	8.3 ± 0.3	24.4 ± 1.0	27.1 ± 1.8	58.3 ± 0.1	241 ± 8	51.2 ± 4.9
Heptane	0.16 ± 0.0	8.6 ± 0.2	18.1 ± 0.7	14.2 ± 3.8	42.5 ± 3.2	170 ± 9	47.6 ± 3.0
Hexane	0.16 ± 0.0	8.4 ± 0.1	15.7 ± 2.9	21.2 ± 3.1	44.1 ± 1.7	180 ± 7	50.1 ± 3.1

- Polyester Content

The fraction of polyester fibres was studied among 9, 11, 13, 15 and 17 wt% of fibres in the dried composites, whose results of bulk density and thermal conductivity are presented in Figure A4.

In terms of thermal conductivity, the best results were achieved with polyester fractions of ~11 wt%, but they were still were higher than expected. The use of heptane as the silylation and washing solvent led to similar density values to those observed in the previous round of experiments. In Figure A4, a trend of lower densities as the fraction of polyester increases is also perceived, analogous to the one previously assessed on cotton-reinforced silica aerogel composites [9].

- Precursor System

It seemed that isoBTES as a co-precursor was the reason for having the not-so-good results, and consequently, other TEOS co-precursors were then tried, namely, vinyltriethoxysilane (VTES) and vinyltrimethoxysilane (VTMS). For these first experiments, the initial volume of 5 mL was kept constant, with 4 mL of TEOS and 1 mL for each co-precursor, thus with slightly different molar ratios. TEOS alone was also used for comparison, as presented in Table A2. The TEOS-VTMS system yielded composites with excellent results, with a thermal conductivity reduced to half the value of that of the TEOS-isoBTES system, at the level of the thermal conductivity of air [82]. The TEOS-VTMS system also provided the lowest density and shrinkage values.

**Table A2:** Structural features and thermal conductivity of the polyester–silica composites manufactured with different precursor systems.

Precursor(s) System	Polyester		Shrinkage (%)			Density (kg m^−3^)	*k* (mW m^−1^ K^−1^)
	(g)	(wt%)	Diameter	Height	Volume		
TEOS	0.23 ± 0.0	11.0 ± 0.4	10.1 ± 0.7	15.7 ± 2.8	31.9 ± 1.3	163 ± 3	32.4 ± 0.8
TEOS-isoBTES	0.23 ± 0.0	11.9 ± 0.7	14.0 ± 0.3	27.5 ± 0.7	46.4 ± 0.9	184 ± 8	47.4 ± 0.7
TEOS-VTES	0.23 ± 0.0	11.5 ± 0.0	8.5 ± 0.1	13.4 ± 0.6	27.6 ± 0.3	145 ± 3	34.6 ± 1.8
TEOS-VTMS	0.23 ± 0.0	10.5 ± 0.3	6.8 ± 0.6	5.6 ± 1.2	18.1 ± 2.0	141 ± 3	25.3 ± 0.3

In spite of the same weight of polyester fibres being used at the beginning of all syntheses, the existing differences in the wt% of the dried composites are related to the losses and release of particles during the silylation and washing steps, that in turn are related to the overall structure and cohesion of the different gels. This can be better understood by observing the appearance of the final products, presented in Figure A5.

- Molar Ratio of Precursor/Co-Precursor

A more detailed study was performed by changing the percentage of the molar ratios of TEOS-VTMS from 65-35 to 85-15. The average densities and thermal conductivities of the composites are presented in Figure A6. The lower value attained in terms of the thermal conductivity is also presented.

The lower value of the average thermal conductivity was achieved at the TEOS-VTMS molar ratio of 80-20. This result is coherent with a work that used the same precursor/co-precursor and yielded a mesoporous structure [83], beneficial for thermal insulation [84]. TEOS is known to favour a structure of mesopores in the silica network [85,86,87], a trend that is outlined in the graph, because the thermal conductivity values decreased with an increasing fraction of TEOS. It is known that the short gelation time is a consequence of fast reaction kinetics, namely, towards the formation of larger pores and a not-so-regular structure [88,89]. In turn, the formation of macropores and inhomogeneous structures leads to an increased porosity and decreased density, which can explain the lowest densities at the TEOS-VTMS percentage molar ratio of 85-15. This reasoning is corroborated by the slightly increased value of the thermal conductivity (also with an increased standard deviation, which can unveil a not-so-consistent silica matrix among all the specimens assessed). This fact may be attributed to the Knudsen effect, because an increased thermal diffusion occurs if there is a higher number of pores with dimensions beyond the minimum free path of air [90].

2. Wool-Reinforced Silica Aerogel Composites

Upon the previous findings discovered on the path to the optimal conditions for cotton- and polyester-reinforced composite development, the optimization of the synthesis of wool-reinforced silica aerogel composites was somehow shortened. The most relevant experiments are described here. In terms of fibre content, the starting point was slightly increased in comparison with cotton, bearing in mind the lower density and the lower thermal conductivity of wool fibres. Another relevant aspect is the lower difference in the thermal conductivity between longitudinal and transversal directions when compared to cotton, a great advantage in the case of reclaimed fibres, because they are curlier and less aligned than the original fibres.

Post-Synthesis Solvent

TEOS-isoBTES was the starting precursor system, by following the optimized stoichiometry and manufacturing conditions of the cotton-reinforced composites, with EtOAc and heptane used as post-synthesis solvents. After the structural assessment and thermal conductivity analyses, there were small differences, yet heptane performed better than EtOAc. However, both in terms of density and thermal conductivity, the results were not as expected, with values superior to 50 mW m^−1^ K^−1^. These results can be seen in Table A3.

**Table A3:** Structural features and thermal conductivity of the first TEOS-isoBTES-based wool–silica aerogel composites, using EtOAc and heptane as the solvents for the silylation and washing steps.

Post-Synthesis Solvent	Wool		Shrinkage (%)			Density (kg m^−3^)	*k* (mW m^−1^ K^−1^)
	(g)	(wt%)	Diameter	Height	Volume		
EtOAc	0.23 ± 0.0	10.6 ± 0.1	11.1 ± 1.1	24.6 ± 2.0	40.4 ± 1.6	175 ± 5	51.4 ± 0.2
Heptane	0.23 ± 0.0	10.4 ± 0.0	11.5 ± 0.8	23.1 ± 2.2	39.8 ± 1.7	169 ± 4	50.1 ± 1.0

- Precursor System

After the excellent results for the polyester-reinforced composites, the VTMS co-precursor was also tried for the wool-reinforced composites, to compare with the isoBTES co-precursor. The volume of precursor (TEOS: 4 mL) and both co-precursors (isoBTES or VTMS: 1 mL) was kept constant. Heptane was the washing silylation solvent, with HMDZ as the silylating agent, as previously. The structural and thermal characterization results are presented in Table A4; the composites can be seen in Figure A7.

**Table A4:** Structural features and thermal conductivity of the wool–silica composites manufactured with different precursor system.

Precursor(s) System	Wool		Shrinkage (%)			Density (kg m^−3^)	*k* (mW m^−1^ K^−1^)
	(g)	(wt%)	Diameter	Height	Volume		
TEOS-isoBTES	0.24 ± 0.0	11.5 ± 0.1	8.7 ± 0.2	13.8 ± 2.5	28.1 ± 2.4	150 ± 3	48.5 ± 1.4
TEOS-VTMS	0.24 ± 0.0	10.7 ± 0.2	5.2 ± 1.1	7.3 ± 0.8	16.6 ± 2.7	136 ± 6	27.2 ± 0.5

The TEOS-VTMS sample exhibited a cohesive structure, in comparison with the TEOS-isoBTES sample, crammed with structural fissures. The comparatively higher wt% of wool fibres of TEOS-isoBTES composites can be explained by the many particles lost during the manufacturing processes. Additionally, the lower shrinkage of the TEOS-VTMS sample led to a lower density and, consequently, low thermal conductivity values, as presented in Table A4.

- Wool Content

In order to find out the optimal amount of wool fibres, the study ranged from 10 to 15 wt%. The lower value of thermal conductivity was attained at the fraction of 11 wt%, as presented in Figure A8, with an excellent performance of 25.7 mW m^−1^ K^−1^.

Similarly to the polyester-reinforced composites, the thermal conductivity decreased slightly from the small content to 11 wt% and increased again in a monotonic way along with the increase in wool content. Nevertheless, the values are nearly stable in this mass interval, and at 15 wt%, a value of 32 mW m^−1^ K^−1^ was achieved for the thermal conductivity, which is still a very good performance. Apart from the aerogel porous structure, the very good insulation of the wool–silica composites can also be attributed to the wool fibre’s inherent crimps, that give rise to the creation of small air pockets. The bulk density values in Figure A8 are lower when compared to the polyester–silica composites, which is attributed to the curly and springy nature of the wool fibres, that led to thicker and airy layers of the carded blanket. Also, there is an almost linear trend of a decrease in bulk density, inversely proportional to the fraction of wool fibre reinforcement.

3. Conclusion of the Optimization Studies for Polyester–Silica and Wool–Silica Composites

After all of the preliminary studies for the manufacturing of the scaled-up samples to test for sound absorption properties, it was decided to proceed with two formulations:

- TEOS-VTMS system, with the molar ratios:
- Si:EtOH:H_2_O:CH_3_COOH:NH_4_OH = 0.82/0.18: 7.79:9.86:4.8 × 10^−2^:9.1 × 10^−2^.

The reasoning behind this choice was to have the same molar ratio of the cotton–silica composites (precursor/co-precursor, TEOS-isoBTES) for a more accurate comparison. Another reason is related to the performance in terms of thermal conductivity, as the molar ratio with the lower values was attained in the previous experiments (see Figure A6). Being a balance between 80-20 and 85-15, the 82-18 molar ratio allows to increase the time of gelation a little bit. It was expected to improve the workability of the procedure, because the gelation time for the TEOS-VTMS molar ratio of 85-15 was less than 3 min.

- TEOS-VTMS System, with the Molar Ratios:

Si:EtOH:H_2_O:CH_3_COOH:NH_4_OH = 0.73/0.27:6.99:6.68:4.4 × 10^−2^:8.2 × 10^−2^.

This is the same recipe of the TEOS-VTMS sample presented in Figure A5 and Figure A7, under which excellent results of thermal conductivity were achieved. This molar ratio also allows a comfortable and more practicable higher gelation time, also being a possible way to create a mesoporous structure, while a higher fraction of VTMS will aid in the build-up of sporadic enlarged pores [91].
